# A systematic review of school health policy measurement tools: implementation determinants and outcomes

**DOI:** 10.1186/s43058-021-00169-y

**Published:** 2021-06-26

**Authors:** Gabriella M. McLoughlin, Peg Allen, Callie Walsh-Bailey, Ross C. Brownson

**Affiliations:** 1 Implementation Science Center for Cancer Control (WU-ISC3) and Prevention Research Center, Brown School, Washington University in St. Louis, Campus Box 1196, One Brookings Drive, St. Louis, MO 63130 USA; 2 Division of Public Health Sciences (Department of Surgery), Washington University School of Medicine, Washington University in St. Louis, St. Louis, 63110 USA

**Keywords:** Dissemination and implementation, Health promotion, Measurement, Policy, Schools

## Abstract

**Background:**

Governments in some countries or states/provinces mandate school-based policies intended to improve the health and well-being of primary and secondary students and in some cases the health of school staff. Examples include mandating a minimum time spent per week in programmed physical activity, mandating provision of healthy foods and limiting fat content of school meals, and banning tobacco products or use on school campuses. Although school health researchers have studied whether schools, districts, or states/provinces are meeting requirements, it is unclear to what extent implementation processes and determinants are assessed. The purposes of the present systematic review of quantitative measures of school policy implementation were to (1) identify quantitative school health policy measurement tools developed to measure implementation at the school, district, or state/provincial levels; (2) describe the policy implementation outcomes and determinants assessed and identify the trends in measurement; and (3) assess pragmatic and psychometric properties of identified implementation measures to understand their quality and suitability for broader application.

**Methods:**

Peer-reviewed journal articles published 1995–2020 were included if they (1) had multiple-item quantitative measures of school policy implementation and (2) addressed overall wellness, tobacco, physical activity, nutrition, obesity prevention, or mental health/bullying/social-emotional learning. The final sample comprised 86 measurement tools from 67 peer-review articles. We extracted study characteristics, such as psychometric and pragmatic measure properties, from included articles based on three frameworks: (1) Implementation Outcomes Framework, (2) Consolidated Framework for Implementation Research, and (3) Policy Implementation Determinants Framework.

**Results:**

Most implementation tools were developed to measure overall wellness policies which combined multiple policy topics (n = 35, 40%) and were in survey form (n = 75, 87%). Fidelity was the most frequently prevalent implementation outcome (n = 70, 81%), followed by adoption (n = 32, 81%). The implementation determinants most assessed were readiness for implementation, including resources (n = 43, 50%), leadership (n = 42, 49%), and policy communication (n = 41, 48%). Overall, measures were low-cost and had easy readability. However, lengthy tools and lack of reported validity/reliability data indicate low transferability.

**Conclusions:**

Implementation science can contribute to more complete and rigorous assessment of school health policy implementation processes, which can improve implementation strategies and ultimately the intended health benefits. Several high-quality measures of implementation determinants and implementation outcomes can be applied to school health policy implementation assessment. Dissemination and implementation science researchers can also benefit from measurement experiences of school health researchers.

**Supplementary Information:**

The online version contains supplementary material available at 10.1186/s43058-021-00169-y.

Contributions to the literature

- This systematic review provides an innovative summary compilation of identified quantitative measures of school health policy implementation determinants and outcomes.
- D&I and school health researchers can benefit from sharing expertise to build an integrated understanding of policy implementation. School health researchers are more familiar with these contexts and can guide contextual assessment, whereas D&I researchers can help guide selection of pre-existing measures and pilot testing of adapted assessment tools.
- Several high-quality measurement tools tested and used in D&I research can be applied in the school setting to inform policy implementation strategies to improve implementation outcomes and ultimately the intended health benefits.
- This review also highlights the need for a focus on health equity as an implementation process and outcome for future study as a means to bridge the gap between policy and practice.

## Introduction

Health policies enacted across multiple levels (e.g., schools, districts, states, countries) are necessary to influence children’s health behaviors [1–6]. Children and adolescents from marginalized communities (e.g., low-income, minoritized racial/ethnic groups) are disproportionately at risk for overweight and obesity, and evidence-based policies present an unmatched opportunity to mitigate social determinants of health [7–9]. Although evidence supports the impact of successful school-based policy implementation on student health outcomes [1, 6, 10–13], the disconnect between evidence-based policy and school-based enactment poses challenges for school administrators and teachers [14]. Therefore, researchers and practitioners have called for enhanced policy implementation research which specifically targets the implementation determinants, processes, and outcomes, in order to enhance the rate at which polices are adopted and infused into organizational culture [14–17].

Specific terms used within dissemination and implementation science (D&I) are implementation determinants, processes, and outcomes [18], which differ from traditional public health research outcomes and offer ways in which researchers can examine how well an innovation is integrated into a particular setting. A clear distinction exists between implementation outcomes and determinants. Implementation outcomes refer to detectable changes in organizational processes and practices as a result of a particular policy or innovation whereas determinants are attributes or characteristics of organizations, innovations, individuals, and the external environment which can be leveraged to increase the likelihood of implementation success [19–22]. Assessment of determinants offers a pragmatic approach to improving implementation efforts since these attributes are dynamic and ever-changing. The Consolidated Framework for Implementation Research (CFIR) [23, 24] represents a means to study implementation determinants. The CFIR comprises five domains which are empirically based influencers of implementation: innovation characteristics (e.g., intervention cost, feasibility, quality), outer setting (e.g., external networks, policies and incentives), inner setting (e.g., readiness, networks, organizational climate/culture), characteristics of individuals (e.g., self-efficacy, motivation), and implementation process (e.g., planning, engaging, executing). Such determinants can be studied to gage what can influence implementation and help to refine implementation efforts over time.

Implementation processes pertain to the specific procedures or practices taking place within a setting to optimize such diffusion. Examples include enforcement of a policy (i.e., “What obligations are there to implement this?”), evaluation (i.e., “What measures are in place to evaluate implementation success?”), and general barriers and facilitators [24, 25]. Finally, implementation outcomes comprise measurable constructs which demonstrate that an implementation effort has been successful, and offer a broader range than fidelity/compliance which facilitates a deeper understanding of context and successful integration [19, 26, 27]. Such concepts have been applied to study policy implementation, with specific applications to public health policy in recent years [28, 29]. Grounded in the model by Proctor et al., implementation outcomes transcend beyond traditional conceptualizations of the research-to-practice paradigm and include adoption, acceptability, appropriateness, cost, fidelity, feasibility, penetration, and sustainability [19]. Measuring multiple implementation outcomes can enhance understanding of how school policies are diffused into practice, and areas for improvement [19, 29]. For example, a school policy may not be perceived as acceptable or appropriate by its stakeholders, which may explain why fidelity and penetration may be lower than anticipated [19]. To date, however, scant literature exists to understand the measures which exist to capture how school-based policies are implemented, warranting further attention to this setting.

One prominent example of school policy is the Child Nutrition and WIC Re-authorization Act [30], which mandated that all schools participating in the National School Lunch Program (NSLP) within the USA develop a comprehensive wellness policy and a plan for implementation. Another example is the Australian New South Wales (NSW) Sport and Physical Activity Policy, which mandates that all children attending primary and secondary schools should participate in a minimum of 150 min of planned moderate activity across the school week [31]. Findings from prior policy/school health promotion implementation research indicate that lack of funding, training/professional development, and administration support are highlighted as key barriers/negative determinants to implementing health promotion policies and programs [32–35], whereas provision of such supports are found to be enabling determinants [32]. However, the measurement tools used to assess implementation outcomes and determinants remain poorly understood [36], thus contributing to the sustained research-practice gap. The overuse and over-dependence of “barriers” and “facilitators” to explain implementation of school health promotion and policy research can contribute to misinformation and to the circulation of highly cited issues (i.e., time, funding, support) [33, 35, 37, 38]. As such, minimal solutions are provided for stakeholders to better implement policies and programs. Furthermore, much of the earlier research has been conducted through qualitative evaluation [1, 10, 39–42], which offers rich information about implementation processes but limits our ability for generalizability and replication.

Although research has examined influential attributes to school health-related policy implementation, matching these attributes to address specific implementation determinants derived through D&I research frameworks [24, 43, 44] will allow for greater use in other school health-related policy topics, and increase the credibility of school-based D&I research and practice. A previous systematic review by Allen et al. [29] investigated quantitative properties of measures of implementation determinants and outcomes pertaining to any type of health policy implemented in clinical or non-clinical settings. The previous review focused on existing measures worded broadly such that they could be applied to study implementation of any health policy type in any setting [29]. Due to such broad focus, however, it was not possible to delve deeper into setting-specific policy implementation measures, limiting the application to school and community-based implementation.

Given the vital role that schools play as a cornerstone of community engagement, understanding how to optimize implementation of health promotion policies can have a significant impact on mitigating health disparities [28]. Advancing this science can provide pragmatic solutions for school researchers and practitioners and optimize the overall impact and sustainability of evidence-based policies. Accordingly, the aims of this systematic review were to (1) identify quantitative school health policy measurement tools developed to measure implementation at the school, district, or state/provincial levels; (2) describe the policy implementation outcomes and determinants assessed and identify the trends in measurement; and (3) assess pragmatic and psychometric properties of identified implementation measures to understand their quality and suitability for broader application.

## Methods

**Fig. 1:**
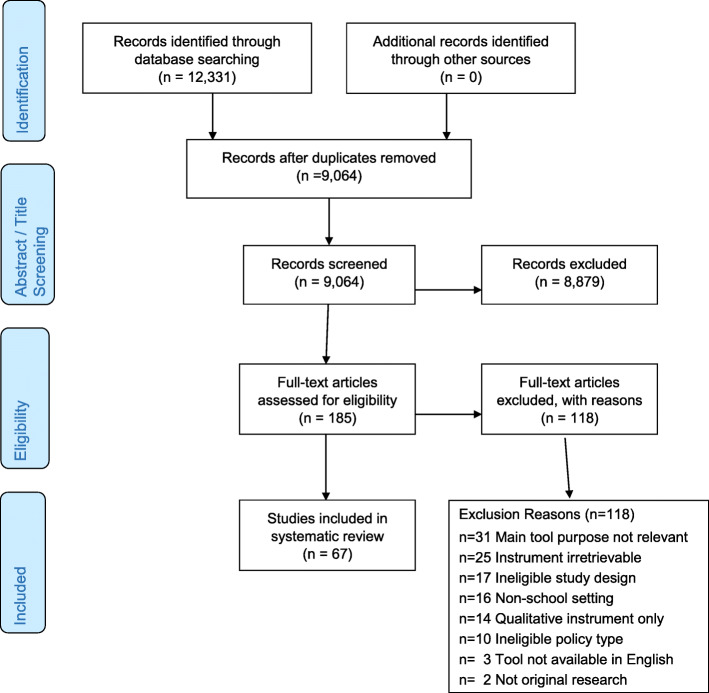
PRISMA chart for systematic review

This review of school-based policy implementation measures was conducted with a similar protocol from the aforementioned Allen et al. review of health policy implementation tools [29]. Both reviews followed procedures for conducting a systematic review of implementation measurement tools [45] and adhered to PRISMA reporting guidelines (see Fig. 1 and Supplemental Table S1) [46]. The review was guided by three D&I frameworks: the Implementation Outcomes Framework (IOF) by Proctor and colleagues [19], the Consolidated Framework for Implementation Research (CFIR) by Damschroder and colleagues to extract implementation determinants [24], and the Policy Implementation Determinants Framework by Bullock and Davis [25, 47]. Through a combination of constructs from these frameworks, we sought to gain a deeper understanding of the implementation outcomes, determinants, and processes for school health policy implementation which are assessed through measurement tools.

The definitions of public policy and policy implementation were standardized to facilitate reliable screening. Specifically, public *policy* includes federal/nation, state/province/county, regional unit, or local level legislation or policies mandated by governmental agencies [48, 49]. The *implementation* of policy conceptualizes the processes by which the mandate is carried out by public or private organizations [49]. For the purpose of this review, the organizations of interest comprised states/provinces, school districts, and primary and secondary pre-university schools as implementing sites.

### Searches

We searched six databases in April 2019 and again in August 2020 to ensure inclusion of recent articles in the present review: MEDLINE, PsycInfo, and CINAHL Plus through EBSCO and PAIS, Worldwide Political, and ERIC through ProQuest. We searched terms at four domains: health, public policy, implementation, and measurement; see Supplemental Table S2 for search terms and syntax. Development of the search strings and terms was based on frameworks in D&I and policy research [29].

### Inclusion and exclusion criteria

The inclusion criteria comprised English-language peer-reviewed journal articles published from January 1995 through August 2020 and utilized quantitative self-report, observational, and/or archival tools to assess implementation of a government-mandated policy [35]. The review by Allen et al. (covering the period 1995–2019) included empiric studies from any continent on policy implementation in any clinical or non-clinical setting on a broad range of health policy topics. Exclusion criteria can be found in Supplementary Table S3. Specific to school settings, we sought articles that met additional criteria: (1) research must have taken place in/with school settings serving students in primary and secondary (ages 5–18; pre-university) schools; (2) measured implementation of school policies already passed or approved that addressed overall wellness, tobacco, physical activity, nutrition, obesity prevention, or mental health/bullying/social-emotional learning; and (3) policy-specific and setting-specific measures were included in the present review but excluded in the initial broad review (which sought generalizable measures that could be applied across multiple settings and topics). In the earlier review by Allen et al. [29], only six instruments that assessed school health policy implementation were worded broadly enough for inclusion in the published paper. The 2019 database searches identified many school health policy implementation measures, but they were excluded from the earlier review as too setting- and policy-specific; hence, the need for this separate more inclusive review of school health policies. Our review included multi-item measures; articles were excluded if the tool included only one relevant item.

### Screening

Two members of the research team used Covidence systematic review software [50] to independently screen all abstracts for inclusion and exclusion. Full texts of all empiric studies of school setting public policy implementation that passed abstract screening in 2019 were rescreened independently in summer 2020 by two coauthors (GMM, PA) for potential inclusion into the present review, with decisions and exclusion reasons coded in Excel. The school setting full-text rescreening was conducted because the Allen review had excluded measures worded specifically for a certain setting or policy topic, whereas such specific measures were included in the present review. The two coauthors also conducted dual independent full-text screening of newly identified 2019–2020 studies that passed abstract screening after the August 2020 updated database searches. The two coauthors met to reach consensus on any inclusion/exclusion disagreements. A third coauthor was consulted if consensus could not be reached. One of the pre-identified exclusion reasons was attributed to each excluded article (for more information see PRISMA chart; Fig. 1).

### Extraction

A comprehensive extraction procedure was implemented in which coauthor (GM, PA, CWB) pairs conducted dual non-independent extraction. A primary reviewer entered relevant information into the extraction database and the secondary reviewer checked data entry for accuracy and completeness. The primary and secondary reviewers then met to reach consensus on any extraction discrepancies; thus final agreement was 100%. Information extracted on the measurement properties included (1) type of measurement tool (i.e., survey, archival, observation), (2) implementation setting (i.e., elementary/primary, middle, high/secondary school, combination of two or more levels), (3) school policy topic (i.e., wellness [two or more health topics], physical activity, nutrition, mental health, tobacco, sun safety), and (4) level of educational entity directing implementation of the governmental mandate (i.e., school, district, state/province, national). Given the broad range of policy topics, we felt it useful to list “wellness policy” as a topic for measures where two or more topics were included in the measurement tool (e.g., physical activity, mental health, nutrition) to avoid over-categorization of measures. Following the three chosen D&I frameworks, all implementation outcomes from the Proctor framework were extracted from measures, followed by selected CFIR constructs which were used in the previous review article and found to be pertinent to policy implementation, and the actor relations/networks and actor context domains from the Bullock and Davis framework. Finally, following the procedures outlined by Lewis and colleagues regarding the Psychometric and Pragmatic Evidence Rating Scale (PAPERS) [45, 51–55], pragmatic (i.e., brevity, cost, readability, training, interpretation) and psychometric (i.e., internal consistency, validity, norms) properties were extracted from each measure to ascertain the quality of each tool. These scoring classifications assign scores from − 1 to 4 based on the degree to which the measures meet each criterion; higher scores on each construct reflect higher quality of the measurement tool (Supplemental Tables S4, S5).

### Data synthesis

Upon achieving consensus on all measures, descriptive analyses were run to gather frequency of items in each school health policy topic. A subset of tools was widely used and/or based on national samples: the Centers for Disease Control and Prevention School Health Policies and Practices Study (school, district, state) [56], the Wellness School Assessment Tool [57], the Maryland Wellness Policies and Practices Project surveys (school and district level) [58], and the Health Enhancing Physical Activity Europe policy audit [59]. We term these “large-scale” tools. Other less frequently reported measures with smaller sample sizes were called “unique tools.” Where appropriate, these measures were analyzed and presented separately when reporting characteristics, given the distinctive differences in methodology and utilization.

## Results

### Aim 1: Elucidate measurement tools used for school health policy implementation

Figure 1 shows the PRISMA flowchart which outlines the steps taken from identifying records through database searching to the studies included in the final review. There were 67 studies included in this review; from these 86 measures were extracted for tool characteristics. From the broad review by Allen et al., six measures from seven studies were also included in in the present review. Of the measures, the vast majority were developed in the USA (n = 60; 69%), followed by Canada (n = 10; 11.6%), European countries (n = 6; 6.9%), and Australia (n = 5; 5.8%). Finally, 2 were developed in India, and 1 each was developed in Indonesia, Mexico, and Taiwan. The 6 studies conducted in Europe were from Denmark (1), the Netherlands (1), Spain (1), or were conducted in multiple countries (3). Table 1 shows the breakdown of tools by school health policy topic and type of tool (i.e., survey, observation, archival). The majority of tools were surveys (n = 75; 87.2%); the most common topic was general wellness policy (i.e., more than two health policy areas; n = 35, 40.6%), followed by nutrition (n = 21; 24.4%) and physical activity (n = 11; 12.7%). Roughly half (n = 42; 49%) of the tool items were generated by experts and 29 measures (33.7%) were piloted with a representative sample. In the included studies, authors reported reliability/validity testing data on pilot testing for 15 measures (17.4%). Of the measures we extracted, psychometric data were available for 28 tools (32.5%).

**Table 1:** Measures by policy topic and type (N = 86)

Wellness topic	Type of measurement tool		
	Archival	Observation	Survey
Health education			2
Mental health			1
Nutrition	1	1	19
Nutrition and physical activity			4
Physical activity	2		9
Sun safety			3
Tobacco/drug			9
Wellness policy	6	1	28
Total	9	2	75

### Aim 2: Investigate implementation determinants and outcomes assessed in the measurement tools

**Fig. 2:**
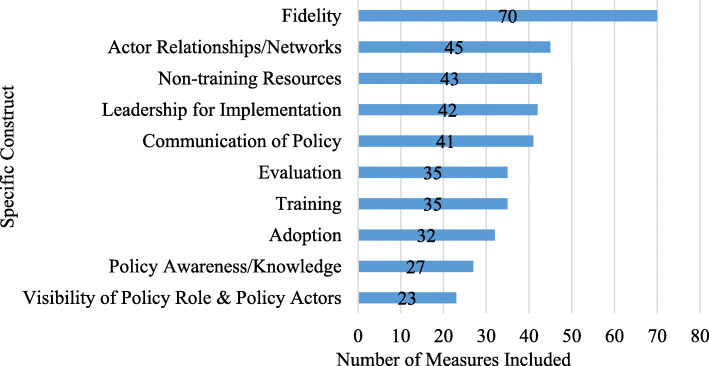
Top 10 most measured constructs of the sample (N = 86)

Table 2 displays the implementation outcomes, processes, and determinants extracted for the overall sample and then shown separately for large-scale tools and unique tools. The most common implementation outcomes assessed were fidelity (n = 70; 81.4%), adoption (n = 32; 37.2%), and acceptability (n = 18; 20.9%). The most prevalent implementation determinants in the sample were actor relations/networks (n = 45; 52.3%), followed by readiness for implementation:non-training resources (n = 43; 50.0%) and leadership for implementation (n = 42; 48.8%). Figure 2 illustrates the most 10 commonly measured constructs for the whole sample. Tools varied in their assessment of fidelity, ranging from asking respondents to report their implementation on a Likert scale, to asking about implementation of multiple “best practices” and reporting frequency of utilization/execution. Adoption typically manifested through asking respondents about their intentions to implement a policy or practice, or about planning activity which has occurred in order for implementation to be successful.

**Table 2:** Implementation outcomes and determinants assessed in measurement tools (N = 86), then split by large-scale and unique tools

Domain		Included measures (N = 86)	%	Large-scale tools (n = 23)	%	Unique tools (n = 63)	%	Definition	Source
Implementation outcomes	Acceptability	18	20.9	0	0.0	18	28.6	Perceptions by staff in organizations mandated to implement the policy, or perceptions of other stakeholders, that the policy mandate is agreeable, palatable, or satisfactory	Proctor et al. [19]
	Adoption	32	37.2	10	43.5	22	34.9	Intention and initial actions of mandated organizations to revise their organizational policies to address policy mandates (not policy development or passage of bills into law)	Proctor et al. [19]
	Appropriateness	9	10.5	0	0.0	9	14.3	Perceived fit, relevance, or compatibility of the [policy] for a given practice setting, provider, or consumer; and/or perceived fit of the [policy] to address a particular issue or problem; context fit	Proctor et al. [19]
	Feasibility	8	9.3	1	4.3	7	11.1	Extent to which a new [policy] can be successfully used or carried out within a given agency or setting; level of administration required to implement a policy, often called policy automaticity	Proctor et al. [19]
	Fidelity/compliance	70	81.4	21	91.3	49	77.8	Degree to which a [policy] was implemented as it was prescribed	Proctor et al. [19]
	Penetration	15	17.4	8	34.8	7	11.1	Integration of a [policy] within a service setting and its subsystems	Proctor et al. [19]
	Sustainability	3	3.5	1	4.3	2	3.2	Extent [new policy] is maintained or institutionalized within a service setting’s ongoing, stable operations	Proctor et al. [19]
	Cost of implementation	5	5.8	0	0.0	5	7.9	Cost impact of an implementation effort	Proctor et al. [19]
Policy/innovation characteristics	Adaptability	3	3.5	0	0.0	3	4.8	Degree to which [a policy] can be adapted, tailored, refined, or reinvented to meet local needs	Damschroder et al. [24]
	Complexity	3	3.5	0	0.0	3	4.8	Perceived difficulty of implementation, reflected by duration, scope, radicalness, disruptiveness, centrality, and intricacy and number of steps required to implement	Damschroder et al. [24]
Organizational characteristics/inner setting	Champions	6	7.0	0	0.0	6	9.5	Field or practice leaders, people who can facilitate and support practice change among professionals	Damschroder et al. [24]
	Organizational culture and climate	9	10.5	1	4.3	8	12.7	Culture: “Norms, values, and basic assumptions of a given organization”; or climate: “Absorptive capacity for change”, extent policy compliance will be rewarded, supported, and expected within their organization	Damschroder et al. [24]; Bullock [47]
	Policy implementation climate (IC)	4	4.7	0	0.0	4	6.3	Organizational climate specific to the policy mandate	Damschroder et al. [24]
	IC: goals and feedback	6	7.0	3	13.0	3	4.8	Degree [the policy mandate] goals are clearly communicated, acted upon, and fed back to staff and alignment of that feedback with goals	Damschroder et al. [24]
	IC: relative priority	21	24.4	2	8.7	19	30.2	Individuals’ shared perception of importance of the [policy] implementation within the organization, competing priorities	Damschroder et al. [24]
	Opinion leaders	7	8.1	0	0.0	7	11.1	Individuals in an organization who have formal or informal influence on attitudes and beliefs of their colleagues with respect to implementing the policy	Damschroder et al. [24]
	Readiness to implement (RI)	5	5.8	0	0.0	5	7.9		Damschroder et al. [24]
	RI: communication of policy	41	47.7	18	78.3	23	36.5	Communication plans and channels created for how the regulatory agency or implementing organization/s will disseminate policy mandate content information to implementers. Actions taken to disseminate policy requirements and guidelines to implementers.	Damschroder et al. [24]
	RI: policy awareness/knowledge	27	31.4	2	8.7	25	39.7	Implementing staff/provider awareness the policy mandate exists, or knowledge of policy content	Damschroder et al. [24]
	RI: leadership for implementation	42	48.8	22	95.7	20	31.7	Commitment, involvement, and accountability of leaders and managers with the implementation	Damschroder et al. [24]
	RI: non-training resources	43	50.0	15	65.2	28	44.4	Level of resources dedicated for implementation and ongoing operations including money…physical space, and time, other than training resources	Damschroder et al. [24]
	RI: training	35	40.7	16	69.6	19	30.2	Training of staff/providers in implementing organizations on how to implement the policy-mandated practices	Damschroder et al. [24]
	Structure of organization	2	2.3	0	0.0	2	3.2	The social architecture, age, maturity, and size of an organization	Damschroder et al. [24]
Implementation process	Enforcement	10	11.6	1	4.3	9	14.3	Strategies used to hold individuals accountable for implementation fidelity/compliance	From screening/coding
	Evaluation	35	40.7	18	78.3	17	27.0	Quantitative and qualitative feedback about the progress and quality of implementation accompanied with regular personal and team debriefing about progress and experience.	Damschroder et al. [24]
	General barriers and facilitators	20	23.3	2	8.7	18	28.6	Factors which facilitate/enable or hinder implementation	From screening/coding
	Collaboration	11	12.8	7	30.4	4	6.3	Active involvement of other stakeholders in the organization to implement the policy	From screening/coding
	Innovation participants	19	22.1	10	43.5	9	14.3	Engaging individuals who will directly benefit/receive the policy action	Damschroder et al. [24]
Actor relationships/networks	Actor relationships/networks	45	52.3	22	95.7	23	36.5	Presence and characteristics of relationships between parallel organizations that must collaborate for policy implementation to be effective	Bullock [47]
	Visibility of policy role and policy actors	23	26.7	8	34.8	15	23.8	Perceived presence and importance of different actors pertinent to implementation of the policy	Bullock [47]
Actor context	Political will for policy implementation	12	14.0	3	13.0	9	14.3	Societal desire and commitment to generate resources to carry out policies	Bullock [47]
	Target population characteristics	1	1.2	0	0.0	1	1.6	Demographics, norms, and neighborhood environments of the population groups that are affecting policy implementation	Bullock [47]
Other domain (not in manual)	CFIR process-planning	2	2.3	0	0.0	2	3.2	The degree to which a scheme or method of behavior and tasks for implementing [a policy] are developed in advance, and the quality of those schemes or methods	Damschroder et al. [24]
	CFIR innovation characteristics-relative advantage	1	1.2	0	0.0	1	1.6	Stakeholders’ perception of the advantage of implementing the intervention versus an alternative solution	Damschroder et al. [24]
	CFIR inner setting-tension for change	1	1.2	0	0.0	1	1.6	The degree to which stakeholders perceive the current situation as intolerable or needing change	Damschroder et al. [24]

For large-scale tools, the most commonly measured determinants were the CFIR readiness for implementation- leadership construct and actor relationships/networks (both n = 22; 95.7%), and the most commonly measured outcome was fidelity from the Proctor model (n = 21; 91.3%). Compared to the whole sample, some constructs which were prevalent in large-scale tools only were the outcome of penetration and the innovation participants determinant from the CFIR-implementation process construct (both n = 8; 34.7%). Among unique tools, fidelity was also the most commonly measured outcome (n = 49; 77.8%) with readiness for implementation-non-training resources (n = 28; 44.4%) as the most common determinant. In terms of least measured constructs, target population characteristics affecting implementation (n = 1) and structure of organization from the CFIR inner setting domain (n = 2) were least measured in the entire sample (see Table 2 for all constructs).

### Aim 3: Evaluate the pragmatic and psychometric properties of measurement tools

**Fig. 3:**
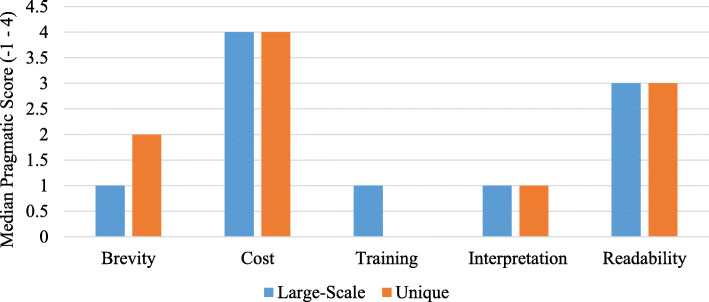
Pragmatic PAPERS scores, by large-scale and unique tools. PAPERS, Psychometric and Pragmatic Evidence Rating Scale [55]

The PAPERS pragmatic scores are shown in Fig. 3 and show separate median scores for the large-scale and unique tools. In terms of brevity, large-scale tools were scored lower as they had a greater number of items (average = 150) compared to unique tools (average = 73). Almost all tools were free or available at very minimal cost to the public (i.e., not required to pay for article and tool if not subscribed to journal), although our team needed to request original items from the corresponding authors for a large proportion of the sample. Large-scale tools scored higher on training for tool administration as most required no/minimal training, compared to unique tools which were often described more ambiguously. However, the unique tools were shorter, provided easier interpretation guidelines, and had lower grade-level reading scores than the larger-scale tools.

Psychometric PAPERS scores were low (0 median) across all components, with large-scale tools generally demonstrating higher quality according to internal consistency and validity (0.66 versus 0.56 mean PAPERS score, out of a possible lowest score of − 1 to a possible highest score of 4). Overall, internal consistency α coefficient scores ranged from 0.40 to 0.98 across the studies. In addition, the sample sizes (2 versus 0.78 mean PAPERS score) used to deduce findings were larger for large-scale studies, ranging from 19 [60] to 6504 schools [61]; samples ranged in between these numbers and were at the student, teacher, school, district, and state/provincial level (see Supplemental Tables S4, S5 for scoring criteria). Very few tool development articles/documents provided concurrent and structural validity information; none of the large-scale tool studies provided such information. Overall, psychometric quality of tools was unknown or low. These results highlight areas for improvement in future tool development and reporting.

Characteristics and PAPERS scores for each tool are provided in Supplementary Table S6. Despite low scores overall, some tools were well-developed and validated according to best practices. One example is the Maryland Wellness Policies and Practices Project (MWPPP) district and school surveys [58], which each received a score of at least 10 (15 for district, 10 for school) for pragmatic and 12 for psychometric properties (Supplementary Table S6). This tool measures overall wellness policy implementation at the school and district levels, assessing multiple implementation outcomes (i.e., adoption, feasibility, fidelity) and determinants (i.e., implementation climate-goals and feedback; readiness-communication of policy, policy awareness/knowledge, leadership, non-training resources, training; actor relationships, visibility of policy role, evaluation, collaboration, innovation participants). This tool may be easily adapted for use within other states and countries depending on policy characteristics. Supplemental Table S6 displays PAPERS scores and tool characteristics for all 86 measured included in the review.

## Discussion

The purpose of this study was to obtain a comprehensive understanding of quantitative implementation measurement tools for school health policy following a systematic review protocol. Findings revealed a large number of tools which covered a wide range of policy topics and implementation settings, with general wellness policy (i.e., two or more health topics) as the most commonly measured area of health promotion. Most of the tools assessing wellness policies more broadly were from the USA, which aligns with federal mandates for schools to develop and implement comprehensive wellness programming [30]. Further, it should be noted that almost all tools were gathered from high-income countries, which draws attention to low- and middle-income countries (LMIC) and the potential for both policy development and implementation evaluation as a means to support ongoing health needs in such populations. Findings from systematic review research highlight a lack of policy/intervention initiatives from LMIC which sought to address child health promotion in the school setting [62]. Accordingly, further work is warranted to examine the fit of existing tools for school contexts in LMIC and to determine how tools from high-income countries may be adapted for use in LMIC to optimize efficiency and sharing of resources.

### Implementation outcomes

Integration of three prominent implementation frameworks in this study facilitated a rich understanding of implementation processes, outcomes, and determinants in a policy context. The finding that fidelity was the most commonly assessed implementation outcome aligns with findings of the broader review by Allen et al. [29], and highlights the high dependence on fidelity as an indicator of implementation success. Several tools only measured fidelity and/or adoption as the implementation outcomes, which draws concern for addressing constructs such as feasibility and sustainability, among others. Only 8 measures addressed feasibility, with 7 of those within unique tools; this is somewhat contradictory to the extant literature on school-based programming, as many studies have reported low feasibility for implementing policies and health promoting interventions [38, 63–65]. Further, through qualitative and mixed-methods research it has transpired that, despite providing financial and logistical support to schools, districts, and states/provinces, most policies are difficult to sustain in absence of such support [65–67]. The finding that only 3 tools measured sustainability is concerning given the emphasis on sustainability/maintenance as a key weakness in implementation science and policy research [19, 68]. Accordingly, it is clear that a greater emphasis on other implementation outcomes and processes would be beneficial in school policy research, given the top-down nature of policy to practice and need to understand how policy and practice can be sustained over time. Measures of implementation outcomes are continuously being developed and tested for validity and reliability, building on earlier work in the education setting [69]. For example, brief measures of acceptability, feasibility, and appropriateness were designed to add in a specific evidence-based practice (or policy) as the item referent; these have preliminary evidence for good reliability and validity [70]. Luke and colleagues developed a measure to assess organizational capacity for sustaining public health and other programs that is reliable and has been tested for construct validity [71]. Although strong examples exist in the healthcare literature [51, 70, 71], there is a need for adaptation and modification to enhance application of D&I within school settings.

Overall, there was a lack of attention paid toward addressing health disparities in the school policy literature and sample of articles. Scholars have stressed the importance of grounding implementation research in health equity principles to examine how implementation efforts may mitigate specific disparities in access to interventions and care [28, 72–74]. Specifically, the Reach, Effectiveness, Adoption, Implementation, Maintenance (RE-AIM) initially developed by Glasgow et al. [68, 75] was adapted to address issues of equity and sustainability with the goal of advancing the science needed to understand how equity can be considered an independent outcome and embedded within each construct to enhance understanding of implementation context [72]. For example, within a school setting it may be useful to measure the community context and sociodemographic characteristics of the school and surrounding community, as a means to understand how implementation of a policy can also promote opportunities for students to engage in health programming, through leveraging community resources [76, 77]. Further research and development is needed in this area to enhance our understanding of health equity and policy implementation.

### Implementation determinants

The finding that readiness for implementation as a general construct was most measured reflects prior research stressing the importance of assessing readiness and organizational capacity for implementation [33, 78–83]. Within this broader construct, non-training resources was the most common determinant assessed; provision of financial resources and personnel support have been cited as supportive factors for policy and innovation implementation in school research [12, 84, 85]. Following this, leadership for implementation was very prevalent in the measures, which again reflects extant knowledge that new innovations require a leader to succeed [65, 86, 87]. Finally, the prevalence of items measuring communication of policy demonstrated the importance of engaging stakeholders in policy implementation through enhancing awareness of such initiatives. Such communication is somewhat understudied as a determinant of implementation in school-based literature according to systematic review research [88, 89] but is perhaps one of the most influential determinants of implementation success. School policy research may be further enhanced by studying the relationships between implementation determinants and outcomes to provide clearer evidence between frameworks such as CFIR and the Proctor outcomes framework [19, 24]. Further, rather than developing completely new measurement tools, those previously tested in community and clinical settings may be used as is or adapted for school settings, facilitating transferability through implementation science [36].

Unlike readiness for implementation, there was a lack of measures to assess the inner setting and implementation process domains, with relative priority (inner setting) and evaluation (implementation process) identified as the most common among constructs. Research has demonstrated the importance of studying organizational culture and climate as a determinant of implementation [86], given that teachers’ actions are encompassed by school- and district-level policies and practices [90, 91]. Some innovations have indeed failed despite leadership for implementation (i.e., small group of leaders taking ownership) due to conflicting organizational practices and lack of priority placed on such initiatives [35, 92]. For true diffusion of innovation to occur, institutional buy-in is essential [93, 94]; future measures development should therefore integrate these constructs as a means to better understand what impacts policy implementation and bridge the research-to-practice gap. Recently, some measures have been developed to address organizational climate and context pertaining to school-based interventions [95]. Such work marks an important step to enhancing implementation measurement within schools; further modification and adaptation is needed to address other implementation determinants, outcomes, and processes.

Finally, as previously mentioned, health equity was absent from determinants measured. As with outcome frameworks, determinant frameworks such as the Health Equity Implementation Framework [73] provide ways to assess implementation context and the structural, sociopolitical, and organizational factors which should be studied to understand how and why implementation occurs in a specific setting. This is particularly salient for schools serving historically marginalized communities such as low-income and communities of color, given the lack of educational funding and support often given to these institutions [33, 96–98]. These factors could and should be studied as the field of policy implementation research grows over time.

### Psychometric and pragmatic properties

Application of the PAPERS rating criteria for pragmatic and psychometric properties revealed areas of strength and need for future improvement [45, 54, 55]. Findings for the pragmatic criteria demonstrated that school policy implementation measures found were generally low-cost and written to a lay audience. However, many tools were long and median scores were driven by large-scale tools such as the SHPPS [56, 99]; a key barrier to conducting research and evaluation with schools is the limited time that stakeholders are able to spend completing surveys and other audit tools, which has implications for data quality and reliability [41, 55, 100, 101]. Although a key need from this study is to adopt pre-existing or develop comprehensive measures which examine implementation outcomes, processes, and determinants, this can lead to lengthy measurement tools which can become arduous to complete and lead to disenfranchisement from stakeholders. Finally, psychometric PAPERS protocols revealed that efforts to ensure quality of tools centered mainly on analyzing internal consistency, with little attention paid to other forms of validity and reliability. This trend is common across other reviews of implementation measures [29, 51] and has implications for broader tool use, specifically when trying to demonstrate implementation efficacy to other populations or policies within school settings. Accordingly, careful tool development should be a focus, and over time it may transpire that some determinants are more influential than others in the policy implementation field, facilitating a streamlined process for subsequent evaluation. Best practices such as field-based pilot testing based on representative samples and developing input from experts are therefore essential in enhancing the pragmatic capabilities of these tools.

### Limitations

Although we conducted a rigorous systematic review following previously established protocols, there are several limitations to note. First, we only extracted tools which were available through online library searches and contacting authors directly where we could not find measures online. We used several approaches to retrieve all tools for extraction, but some tools were unavailable online or from the study authors. We were unable to analyze tools for which we could not access original items. We did not conduct citation searching to find all empiric uses of each included measure, so we may not have captured all adaptations of each measure. Tools from the grey literature were also not included in this review; although we searched for manuals and tools available online, it was required they were cited in a peer-reviewed article first. Second, we did not explicitly screen for health equity constructs, but based on our review of included tools there was not much to be gleaned in terms of health equity and policy. Several implementation science frameworks integrate health equity and these help to provide guidance for future measurement development [72, 73, 102–106]. Finally, although we took a comprehensive policy approach, some policy topics were excluded (i.e., not directly related to health/wellness topics), and in excluding these we may have overlooked other pertinent measurement tools.

## Conclusions

What gets measured gets achieved [107] — our review suggests that more comprehensive measurement tools are needed for school policy research that come from or could potentially be transferred to other settings (i.e., community, clinical). Enhancing the quality of policy D&I research through high-quality pragmatic measures will mark a key step in bridging the policy to practice gap [52, 75, 108]. Future assessment of implementation of policies intended to improve school staff well-being is also needed. Furthermore, given the lack of focus on addressing health equity, there is now an opportunity to apply or develop tools which can help distinguish practices that address health disparities. The WIC Child Nutrition Re-Authorization act [30] and USDA Healthy Hunger Free Kids Act (HHFKA) [109] are examples of policies which inherently are aimed at reducing health inequality given the focus on NSLP integration, but we know little about how their implementation may influence social determinants of health. Thus, more explicitly addressing health equity is a priority for future research and practice in health policy, in order to elicit a meaningful impact on population health.

## Supplementary Information

**Additional file 1: Supplemental Table S1**. PRISMA 2009 checklist. **Supplemental Table S2**. Electronic database search terms. **Supplemental Table S3**. Inclusion and exclusion criteria. **Supplemental Table S4**. Psychometric and Pragmatic Evidence Rating Scale (PAPERS) Pragmatic rating scales. **Supplemental Table S5**. Psychometric and Pragmatic Evidence Rating Scale (PAPERS) Psychometric rating scales. **Supplemental Table S6**. Measures Information and Psychometric and Pragmatic Evidence Rating Scale (PAPERS) Scores.

## Data Availability

A compendium of identified measures is publicly available for dissemination at https://www.health-policy-measures.org/. A link is provided on the website of the Prevention Research Center, Brown School, Washington University, in St. Louis, at https://prcstl.wustl.edu/. The authors invite interested organizations to provide a link to the compendium on their own websites. Citations and abstracts of excluded policy-specific measures are available on request.
