# Supplementary material for: A systematic review of school health policy measurement tools: implementation determinants and outcomes
Source: Implement Sci Commun. 2021 Jun 26;2:67. doi: 10.1186/s43058-021-00169-y (PMC8235584; doi:10.1186/s43058-021-00169-y)
Supplement: Supplementary file 1 — Additional file 1: Supplemental Table S1. PRISMA 2009 checklist. Supplemental Table S2. Electronic database search terms. Supplemental Table S3. Inclusion and exclusion criteria. Supplemental Table S4. Psychometric and Pragmatic Evidence Rating Scale (PAPERS) Pragmatic rating scales. Supplemental Table S5. Psychometric and Pragmatic Evidence Rating Scale (PAPERS) Psychometric rating scales. Supplemental Table S6. Measures Information and Psychometric and Pragmatic Evidence Rating Scale (PAPERS) Scores. [file 43058_2021_169_MOESM1_ESM.docx]

**SUPPLEMENTAL TABLES**

Supplemental Table S1. PRISMA 2009 checklist.

Supplemental Table S2. Electronic database search terms.

Supplemental Table S3. Inclusion and exclusion criteria.

Supplemental Table S4. Psychometric and Pragmatic Evidence Rating Scale (PAPERS) Pragmatic rating scales

Supplemental Table S5. Psychometric and Pragmatic Evidence Rating Scale (PAPERS) psychometric rating scales.

Supplemental Table S6. Measures Information and Psychometric and Pragmatic Evidence Rating Scale (PAPERS) Scores.

**Supplemental Table S1. PRISMA 2009 checklist.**

| **Section/topic** | **#** |  | | **Checklist item** | **Reported on page #** |
| --- | --- | --- | --- | --- | --- |
|  | | | **TITLE** | |  |
| Title | 1 |  | | Identify the report as a literature review. | 1 |
|  | | | **ABSTRACT** | |  |
| Structured summary | 2 |  | | Provide a structured summary including, as applicable: background; objectives; data sources; study eligibility criteria, participants, and interventions; study appraisal and synthesis methods; results; limitations; conclusions and implications of key findings; | 2 |
|  | | | **INTRODUCTION** | |  |
| Rationale | 3 |  | | Describe the rationale for the review in the context of what is already known about your topic. | 5 |
| Objectives | 4 |  | | Provide an explicit statement of questions being addressed with reference to participants, interventions, comparisons, outcomes, and study design (PICOS). | 8 |
|  | | | **METHODS** | |  |
| Eligibility criteria | 5 |  | | Specify study characteristics (e.g., PICOS, length of follow-up) and report characteristics (e.g., years considered, language, publication status) used as criteria for eligibility, giving rationale. | 10 |
| Information sources | 6 |  | | Describe all information sources (e.g., databases with dates of coverage) in the search and date last searched. | 9 |
| Search | 7 |  | | Present full electronic search strategy for at least one database, including any limits used, such that it could be repeated. | 10 |
| Study selection | 8 |  | | State the process for selecting studies (i.e., screening, eligibility). | 10 |
| Risk of bias in individual studies | 9 |  | | Describe methods used for assessing risk of bias of individual studies (including specification of whether this was done at the study or outcome level). | 10-11 |
| Risk of bias across studies | 10 |  | | Specify any assessment of risk of bias that may affect the cumulative evidence (e.g., publication bias, selective reporting within studies). | NA |
|  | | | **RESULTS** | |  |
| Study selection | 11 |  | | Give numbers of studies screened, assessed for eligibility, and included in the review, with reasons for exclusions at each stage, ideally with a flow diagram. | 13 |
| Study characteristics | 12 |  | | For each study, present characteristics for which data were extracted (e.g., study size, PICOS, follow-up period) and provide the citations. | 13 |
| Synthesis of results of individual studies | 13 |  | | For all outcomes considered (benefits or harms), present, for each study: (a) summary of results and (b) relationship to other studies under review (e.g. agreements or disagreements in methods, sampling, data collection or findings). | 13-16 |
|  | | | **DISCUSSION** | |  |
| Summary of evidence | 14 |  | | Summarize the main findings including the strength of evidence for each main outcome; consider their relevance to key groups (e.g., healthcare providers, users, and policy makers). | 16-21 |
| Limitations | 15 |  | | Discuss limitations at study and outcome level (e.g., risk of bias), and at review-level (e.g., incomplete retrieval of identified research, reporting bias). | 21 |
|  | | | **CONCLUSION** | |  |
| Conclusions | 16 |  | | Provide a general interpretation of the results in the context of other evidence, and implications for future research. | 22 |

**Supplemental Table S2. Electronic database search terms.**

| String  Search Label | String  Topic | Search Terms |
| --- | --- | --- |
| S1  TI OR AB OR SU | Health | "health" OR "healthcare" OR "healthy" OR "healthier" OR "wellness" |
| S2  TI OR AB OR SU | Public Policy | "policy" OR "policies" OR "law" OR "laws" OR "legislation" OR "legislative" OR "statute" OR "statutes" OR "regulation" OR "regulations" OR "regulatory" OR "executive order" OR "executive orders" OR "congress" OR "congresses" OR "congressional" OR "city council" OR "city councils" OR "county council" OR "county councils" OR mandat* OR "ordinance" OR "ordinances" OR "rule" OR "rules" |
| S3  TI OR AB OR SU | Implementation | “implement*” OR disseminat* OR "institutionalization" OR "institutionalisation" OR "integrate" OR "integrates" OR "integrated" OR "integrating" OR "integration" OR "integrations" OR "knowledge transfer" OR "knowledge exchange" OR "knowledge translation" OR "knowledge diffusion" OR “knowledge utilization” OR "research utilization" OR "innovation" |
| S4  TI OR AB OR SU | Measurement | "measure" OR "measures" OR "measurement" OR "measurements" OR "instrument" OR "instruments" OR "survey" OR "surveys" OR "questionnaire" OR "questionnaires" OR "scale" OR "scales" OR "self-report" OR "self-reports" OR "self-reported" OR "archived data" OR “archival data” OR "quantitative" OR "quantitatively" OR "inventory" OR "inventories" OR "rating" OR "ratings" OR "assessment form" OR "assessment forms" OR "evaluation form" OR "evaluation forms" OR "tool" OR "tools" OR "index" OR "indexes" OR "indices" |
| Syntax: Row 1: (S1 AND S2 AND S3 AND S4)  Row 2: AND (S5 OR S6 OR S7 OR S8) | | |
| S5  TI OR AB | Policy near implementation | “policy” N7 (implement* OR “adoption” OR "institutionalization" OR "institutionalisation" OR "integrate" OR "integrates" OR "integrated" OR "integrating" OR "integration" OR "integrations" OR "knowledge transfer" OR "knowledge exchange" OR "knowledge translation" OR "knowledge diffusion" OR “knowledge utilization” OR "research utilization" OR "innovation") |
| S6  TI OR AB | Policies near implementation | “policies” N7 (implement* OR “adoption” OR "institutionalization" OR "institutionalisation" OR "integrate" OR "integrates" OR "integrated" OR "integrating" OR "integration" OR "integrations" OR "knowledge transfer" OR "knowledge exchange" OR "knowledge translation" OR "knowledge diffusion" OR “knowledge utilization” OR "research utilization" OR "innovation") |
| S7  TI OR AB | Mandate near implementation | “policies” N7 (implement* OR “adoption” OR "institutionalization" OR "institutionalisation" OR "integrate" OR "integrates" OR "integrated" OR "integrating" OR "integration" OR "integrations" OR "knowledge transfer" OR "knowledge exchange" OR "knowledge translation" OR "knowledge diffusion" OR “knowledge utilization” OR "research utilization" OR "innovation") |
| S8  TI OR AB | Regulation near implementation | “regulation” N7 (implement* OR “adoption” OR "institutionalization" OR "institutionalisation" OR "integrate" OR "integrates" OR "integrated" OR "integrating" OR "integration" OR "integrations" OR "knowledge transfer" OR "knowledge exchange" OR "knowledge translation" OR "knowledge diffusion" OR “knowledge utilization” OR "research utilization" OR "innovation") |
| Additional syntax used in the updated search August 4, 2020 to identify articles published 2019-2020 not captured in the original April 4 2019 searches: | | |
| S9 | Chronic disease – risk factors | “obesity” OR “obese” OR “overweight” OR “weight” OR “BMI” OR “body mass index” OR “exercise” OR “physical activity” OR “physical inactivity” OR “physical fitness” OR “sedentary” OR “lifestyle” OR “diet*” OR “dietary intake” OR “food intake” OR “healthy eating” OR nutrition* OR “fruit*” OR “vegetable*” OR “food*” OR ”soft drink*” OR “soda” OR “sweetened” OR sugar* OR smok* OR “smoking cessation*” OR “tobacco” OR “cigarette” OR nicotine OR vape* OR “vaping” OR “e-cig*” OR “electronic cigarette” OR “pipe” OR “cigar” OR “hookah” OR “alcohol*” OR “drink*” OR “drunk*” OR “liquor*” OR “intoxicat*” |

Notes:

Databases searched via EBSCO: CINAHL Plus, Medline, PsychInfo;

Databases searched via ProQuest with slightly different syntax: PAIS Index, Worldwide Political Science Abstract, ERIC

Search terms in Title (TI), Abstract (AB) or Subject Headings (SU)

Limiters: 1995 forward, Academic articles, English language

**Supplemental Table S3. Inclusion and Exclusion Criteria.**

|  | Include | Exclude |
| --- | --- | --- |
| Year Published | 1995 or later | Before 1995 |
| Language | English | Non-English |
| Country | US, Australia, Canada, Europe, New Zealand & other democratically-governed countries | Non-democratically governed countries  World-wide studies |
| Setting | - Organizations preparing for policy implementation, target organizations institutionalizing the policy, enforcement organizations - Schools: only K-12 school settings which can be public, charter, or private schools. | - If only target population or organizations that advocated for bill/policy passage - Mental health or residential clinical settings: rehabilitation centers, social work, psychiatric care, residential facilities, community mental health providers - Daycare or college settings where the affected population is not between PreK-12 grade levels |
| Study Design | - Empirical quantitative (or mixed quantitative and qualitative) studies   Contains original or secondary data collection   - Case studies or protocol w/measures - Longitudinal - Cross-sectional | - If only study health outcomes of policy (target population changes health behavior or status) - Conceptual, editorials, commentaries, narrative reviews or systematic review (use only to find articles), books, theses, non-peer reviewed documents - Bill analyses/policy content analyses without implementation assessment - No original or secondary data collection |
| Measurement Type | - Quantitative self-report survey measures - Quantitative archival measures - Mixed quantitative and qualitative studies | - Qualitative-only (e.g. If only open-ended interview guide or observation checklist) - Cost only or other economic measurement studies |
| Measures Assess Implementation of: | - Implementation of school policies already passed or approved that addressed overall wellness, tobacco, physical activity, nutrition, obesity prevention, and mental health/bullying/social-emotional learning. | - Social policy implementation (e.g. housing) - Conceptual studies about implementation constructs - Quantitative measurement of individual-level health outcome (e.g. tobacco cessation rates post-policy) - Analysis of policy content - Policy development - Policy related to workforce development (e.g. licensure) - Environmental public policy without explicit mention of physical or mental health |
| Measurement property domains assessed | Required so can assess pragmatic measurement properties (Glasgow & Riley, 2013; Glasgow 2013, Lewis2018):   - Quantitative measures - Provides number of items (for brevity rating) - Provides item wording or source article reference w/item wording (for language level)   Desirable to report:   - Reliability - Validity - How to score and how to interpret scores (for part of feasibility/low burden rating) - Sensitivity to change (suitability for repeated administration) - Connection to theory or model - How others can use data (actionability) - Importance to practitioners |  |

**Supplemental Table S4. Psychometric and Pragmatic Evidence Rating Scale (PAPERS) Pragmatic rating scales.**

| **Brevity (length)** | |
| --- | --- |
| **-1** | Poor (P): The measure has greater than 200 items. |
| **0** | None (N): The measure is not available for use in the public domain. |
| **1** | Minimal/Emerging (M): The measure has greater than 100 items but fewer than *or equal to 200 items.* |
| **2** | Adequate (A): The measure has greater than 50 items but fewer than *or equal to 100 items.* |
| **3** | Good (G): The measure has greater than 10 items but fewer than *or equal to 50 items.* |
| **4** | Excellent (E): The measure has fewer than or equal to *10 items.* |
| **Cost** | |
| **-1** | Poor (P): The measure is extremely costly greater than or equal to $100 per use |
| **0** | None (N): The cost of the measure is unknown |
| **1** | Minimal/Emerging (M): The measure is very costly greater than or equal to $50 but < $100 per use |
| **2** | Adequate (A): The measure is somewhat costly greater than or equal to $1 but < $50 per use |
| **3** | Good (G): The measure is not costly < $1 per use |
| **4** | Excellent (E): The measure is free and in the public domain |
| **Assessor Burden (ease of training)** | |
| **-1** | Poor (P): The measure requires an external, expert administrator, with no option to self-train or for a train-the-administrator component. |
| **0** | None (N): The training and administration information for the measure is unavailable. |
| **1** | Minimal/Emerging (M): The measure requires a train-the-trainer to administer component that is specialized or includes a significant cost (greater than or equal to $100). |
| **2** | Adequate (A): The measure requires some training, in addition to a manual, and/or supervision/consultation with experts is needed to administer the measure which includes minimal cost (i.e., small consultant fee) (greater than or equal to $50 but less than $100) |
| **3** | Good (G): The measure includes a manual in order to self-train for administration and the cost for the manual is free or minimal (less than $50 but not free). |
| **4** | Excellent (E): The measure requires no training and/or has free automated administration |
| **Assessor Burden (easy of interpretation)** | |
| **-1** | Poor (P): The measure requires an expert to score and interpret, though no entity to whom to send the measure is identified, and no information on handling missing data is provided |
| **0** | None (N): The ease of interpreting the measure cannot be assessed because the measure is not in the public domain |
| **1** | Minimal/Emerging (M): The measure does not include suggestions for interpreting score ranges, no clear cut-off scores, and no instructions for handling missing data |
| **2** | Adequate (A): The measure includes a range of scores with few suggestions for interpreting them but no clear cut-off scores and no instructions for handling missing data |
| **3** | Good (G): The measure includes a range of scores with value labels and cut-off scores, but scoring requires manual calculation and/or additional inspection of response patterns or subscales, and no instructions for handling missing data are provided |
| **4** | Excellent (E): The measure includes clear cut-off scores with value labels, instructions for handling missing data are provided, and calculation of scores is automated or scores can be sent off to an identified entity for calculation with results returned |
| **Language** | |
| **-1** | Poor (P): The measure used language that was only readable by experts in its content |
| **0** | None (N): The measure was not available in the public domain and therefore the readability cannot be assessed |
| **1** | Minimal/Emerging (M): The readability of the measure was at a graduate study level (range: 17.0 and above). |
| **2** | Adequate (A): The readability of the measure was at a college level (range: 13.0 – 16.99). |
| **3** | Good (G): The readability of the measure was between an 8th and 12th grade level (range: 8.0 – 12.99). |
| **4** | Excellent (E): The readability of the measure was at or below an 8th grade level (range: 7.9 and below) |

**Sources:**

1. Henrikson NB, Blasi PR, Dorsey CN, Mettert KD, Nguyen MB, Walsh-Bailey C, et al. Psychometric and Pragmatic Properties of Social Risk Screening Tools: A Systematic Review. Am J Prev Med. 2019;57(6S1):S13-S24.

2. Lewis CC, Mettert KD, Dorsey CN, Martinez RG, Weiner BJ, Nolen E, et al. An updated protocol for a systematic review of implementation-related measures. Syst Rev. 2018;7(1):66.

3. Powell BJ, Stanick CF, Halko HM, Dorsey CN, Weiner BJ, Barwick MA, et al. Toward criteria for pragmatic measurement in implementation research and practice: a stakeholder-driven approach using concept mapping. Implement Sci. 2017;12(1):118.

4. Stanick CF, Halko HM, Nolen EA, Powell BJ, Dorsey CN, Mettert KD, et al. Pragmatic measures for implementation research: development of the Psychometric and Pragmatic Evidence Rating Scale (PAPERS). Translational Behavioral Medicine. 2019.

5. Glasgow RE. What does it mean to be pragmatic? Pragmatic methods, measures, and models to facilitate research translation. Health Educ Behav. 2013;40(3):257-65.

**Supplemental Table S5. Psychometric and Pragmatic Evidence Rating Scale (PAPERS) psychometric rating scales.**

| **Reliability - Internal Consistency** | |
| --- | --- |
| -1 | Poor (P): Cronbach’s α values of **< 0.50** |
| 0 | None (N): Internal consistency measures are not applicable for this instrument **OR** classical test theory anchors are not appropriate (results reported using item response theory) **OR** α values are not yet available for the full measure scale or any associated subscales. |
| 1 | Minimal/Emerging (M): Cronbach’s α values = **0.50-0.69** |
| 2 | Adequate (A): Cronbach’s α values of = **0.70 - 0.79** |
| 3 | Good (G): Cronbach’s α values of = **0.80 - 0.89** |
| 4 | Excellent (E): Cronbach’s α values of **≥ 0.90** |
| **Norms** | |
| -1 | Poor (P): Measures of central tendency and distribution for the total score (and subscales if relevant) based only on a very small (**n < 50**) sample are available. |
| 0 | None (N): Norms not yet available. |
| 1 | Minimal/Emerging (M): Measures of central tendency and distribution for the total score (and subscales if relevant) based only on a small (**n = 50-99**) sample are available. |
| 2 | Adequate (A): Measures of central tendency and distribution for the total score (and subscales if relevant) based only on a small (**n = 100-299**) sample are available. |
| 3 | Good (G): Measures of central tendency and distribution for the total score (and subscales if relevant) based on a medium (**n = 300-499**) sample are available. |
| 4 | Excellent (E): Measures of central tendency and distribution for the total score (and subscales if relevant) based on a large (**n ≥ 500**) sample are available. |
| **Construct Validity - Convergent** | |
| -1 | Poor: **Cohen’s *d* ≤ 0.10** |
| 0 | None (N): Convergent validity measures are not applicable for this instrument OR convergent validity was not assessed. |
| 1 | Minimal/Emerging: **0.10 < Cohen’s *d* ≤ 0.20** |
| 2 | Adequate: **0.20 < Cohen’s *d* ≤ 0.50** |
| 3 | Good: **0.50 < Cohen’s *d* ≤ 0.80** |
| 4 | Excellent: **Cohen’s *d* > 0.80** |
| **Construct Validity - Divergent** | |
| -1 | Poor: **Cohen’s *d* > 0.80** |
| 0 | None (N): Discriminant validity measures are not applicable for this instrument OR discriminant validity was not assessed. |
| 1 | Minimal/Emerging: **0.50 < Cohen’s *d* ≤ 0.80** |
| 2 | Adequate: **0.20 < Cohen’s *d* ≤ 0.50** |
| 3 | Good: **0.10 < Cohen’s *d* ≤ 0.20** |
| 4 | Excellent: **Cohen’s *d* ≤ 0.10** |
| **Construct Validity – Known-Groups** | |
| -1 | Poor (P): Known-groups validity failed to be detected. |
| 0 | None (N): Known-groups validity not yet tested. |
| 1 | Minimal/Emerging (M): Statistically significant difference between groups detected, but no hypothesis tested |
| 2 | Adequate (A): Two or more statistically significant difference between groups detected, but no hypotheses tested |
| 3 | Good (G): Statistically significant difference between groups detected AND hypothesis tested |
| 4 | Excellent (E): Two or more statistically significant differences between groups detected AND hypotheses tested |
| **Criterion Validity - Predictive** | |
| Evidence of correlation (Pearson’s r) between instrument and scores on another test (measuring a distinct construct of interest or outcome) administered at some point in the future. | |
| -1 |  |
| 0 | None (N): Predictive validity not tested. |
| 1 | Minimal/Emerging (M): Pearson’s *r* = **0.10-0.29** |
| 2 | Adequate (A): Pearson’s *r* = **0.30-0.49** |
| 3 | Good (G): Pearson’s *r* = **0.50-0.69** |
| 4 | Excellent (E): Pearson’s *r* **> 0.70** |
| **Criterion Validity - Concurrent** | |
| -1 | Poor (P): Pearson’s *r* **< 0.10** |
| 0 | None (N): Concurrent validity not tested. |
| 1 | Minimal/Emerging (M): Pearson’s *r* = **0.10-0.29** |
| 2 | Adequate (A): Pearson’s *r* = **0.30-0.49** |
| 3 | Good (G): Pearson’s *r* = **0.50-0.69** |
| 4 | Excellent (E): Pearson’s *r* **> 0.70** |
| **Dimensionality – Structural Validity** | |
| Normed Fit Index = NFI ; Incremental Fit Index = IFI ; Goodness of Fit Index = GFI ; Tucker-Lewis Index = TLI; Comparative Fit Index = CFI ; Relative Noncentrality Fit Index = RNI; Standardized RMR = SRMR ; Root Mean Square Error of Approximation = RMSEA; Weighted Root Mean Residual = WRMR | |
| -1 | Poor (P): The sample consisted of less than 5 times the number of items AND exploratory factor analysis explained **< 25%** of variance **OR** |
|  | NFI **OR** IFI **OR** GFI **OR** TLI **OR** CFI **OR** RNI  **≤ 0.88 OR** SRMR **OR** RMSEA = **X ≥ 0.10 OR** WRMR **≥ 0.92** |
| 0 | None (N): No exploratory or confirmatory factor analysis has yet been performed, nor have any Item Response Theory (IRT) tests of (uni-) dimensionality have been conducted **OR** analysis has been conducted but percent variance is unexplained and cannot be calculated **OR** only principal components analysis has been conducted. |
| 1 | Minimal/Emerging (M): The sample consisted of 5 times the number of items AND exploratory factor analysis explained **< 25%** of variance **OR** |
|  | NFI **OR** IFI **OR** GFI **OR** TLI **OR** CFI **OR** RNI = **0.88 < X ≤ 0.90 OR** SRMR **OR** RMSEA = **0.08 ≤ X < 0.10 OR** WRMR = **0.90 ≤ X < 0.92** |
| 2 | Adequate (A): The sample consisted of 5 times the number of items but is less than 100 in total AND an exploratory factor analysis explained **< 50%** of variance **OR** |
|  | NFI **OR** IFI **OR** GFI **OR** TLI **OR** CFI **OR** RNI = **0.90 < X ≤ 0.95 OR** SRMR **OR** RMSEA = **0.05 ≤ X < 0.08 OR** WRMR = **0.85 ≤ X < 0.90** |
| 3 | Good (G): The sample consisted of 5 times the number of items and is greater than or equal to 100 in total OR the sample consisted of 5-7 times the number of items but is less than 100 in total AND in either case exploratory factor analysis explained  **< 50%** of variance **OR** |
|  | NFI **OR** IFI **OR** GFI **OR** TLI **OR** CFI **OR** RNI = **0.95 < X ≤ 0.97 OR** SRMR **OR** RMSEA = **0.03 ≤ X < 0.05 OR** WRMR = **0.83 ≤ X < 0.85** |
| 4 | Excellent (E): The sample consisted of 7 times the number of items and is greater than 100 in total AND an exploratory factor analysis explained **> 50%** of variance **OR** |
|  | NFI **OR** IFI **OR** GFI **OR** TLI **OR** CFI **OR** RNI  **> 0.97 OR** SRMR **OR** RMSEA = **< 0.03 OR** WRMR **< 0.83** |
| **Responsiveness** | |
| Standardized Response Mean = SRM | |
| -1 | Poor (P): SRM **< 0.10 OR** Pearson’s *r* **< 0.10** |
| 0 | None (N): The instrument has not been administered both pre- and post- implementation to evaluate sensitivity to change. |
| 1 | Minimal/Emerging (M): SRM = **0.10-0.19 OR** Pearson’s *r* = **0.10-0.29** |
| 2 | Adequate (A): SRM = **0.20-0.49 OR** Pearson’s *r* = **0.30-0.49** |
| 3 | Good (G): SRM = **0.50-0.79 OR** Pearson’s *r* = **0.50-0.69** |
| 4 | Excellent (E): SRM **> 0.80 OR** Pearson’s *r* **> 0.70** |

Henrikson NB, Blasi PR, Dorsey CN, Mettert KD, Nguyen MB, Walsh-Bailey C, et al. Psychometric and Pragmatic Properties of Social Risk Screening Tools: A Systematic Review. Am J Prev Med. 2019;57(6S1):S13-S24.

Lewis CC, Mettert KD, Dorsey CN, Martinez RG, Weiner BJ, Nolen E, et al. An updated protocol for a systematic review of implementation-related measures. Syst Rev. 2018;7(1):66.

**Supplemental Table S6: Measures Information and Psychometric and Pragmatic Evidence Rating Scale (PAPERS) Scores.**

| **Large-Scale Tools (N=23)** |  |  |  |  |  |  |  |
| --- | --- | --- | --- | --- | --- | --- | --- |
| **Tool Name** | **Year of Development** | **Policy Topic** | **Number of Items** | **Developed by (Author)** | **Setting** | **PAPERS Pragmatic** | **PAPERS Psychometric** |
| School Health Policies and Practices Survey- State Survey | 2012 | Health Education | 182 | Centers for Disease Control (CDC) | States | 13 | 0 |
|  |  | Mental Health | 76 |  | States | 14 | 0 |
|  |  | Nutrition | 127 |  | States | 13 | 5 |
|  |  | Physical Activity | 166 |  | States | 13 | 1 |
|  | 2006 | Wellness Policy/Environment | 125 |  | States | 8 | 1 |
| School Health Policies and Practices Survey- District Survey | 2006 | Nutrition | 175 | Centers for Disease Control (CDC) | School Districts | 8 | 4 |
|  |  | Physical Activity | 200 |  | School Districts | 8 | 4 |
|  |  | Wellness Policy/Environment | 272 |  | School Districts | 6 | 4 |
| School Health Policies and Practices Survey- School Survey | 2006 | 2006 Wellness Policy/Environment | 307 | Centers for Disease Control (CDC) | Schools (all levels) | 6 | 4 |
|  |  | 2006 Physical Education | 182 |  | Schools (all levels) | 8 | 4 |
|  | 2012 | 2012 Wellness Policy/Environment | 227 |  | Schools (all levels) | 6 | 4 |
|  |  | 2012 Physical Education | 278 |  | Schools (all levels) | 6 | 4 |
|  |  | 2012 Nutrition | 243 |  | Schools (all levels) | 7 | 4 |
| Wellness School Assessment Tool | 2009 | Wellness Policy | 96 | Schwartz et al. (Rudd Center) | School Districts | 11 | 2 |
| Maryland Wellness Policies and Practices Project - District Survey | 2013 | Wellness Policy | 104 | Hager et al. | School Districts | 15 | 12 |
| Maryland Wellness Policies and Practices Project - School Survey | 2013 | Wellness Policy | 97 | Hager et al. | Schools (all levels) | 10 | 12 |
|  | 2015 | Wellness Policy | 56 |  | Schools (all levels) | 10 | 12 |
|  | 2017 | Wellness Policy | 98 |  | Schools (all levels) | 10 | 12 |
| Healthy Eating and Physical Activity (HEPA) Europe Survey | 2014 | Nutrition and Physical Activity | 75 | Bull et al. | Schools (all levels) | 10 | 0 |
| Healthy Eating and Physical Activity (HEPA) Europe Survey Version 2 | 2015 | Nutrition and Physical Activity | 75 |  | Schools (all levels) | 10 | 0 |
|  |  |  |  |  |  |  |  |
| **Unique Tools (n=63)** |  |  |  |  |  |  |  |
| **Tool Name** | **Year of Development** | **Policy Topic** | **Number of Items** | **Developed by (Author)** | **Setting** | **PAPERS Pragmatic (Total)** | **PAPERS Psychometric (Total)** |
| Abbott Physical Activity Facilitator Survey | 2011 | Physical Activity | NR | Abbott, Rebecca | ES | 10 | 0 |
| Daily Physical Activity Policy -Principal survey | 2016 | Physical Activity | 76 | Allison, Kenneth | ES | 10 | 4 |
| Daily Physical Activity Policy -Teacher survey | 2016 | Physical Activity | 57 | Allison, Kenneth | ES | 10 | 4 |
| Patel 2020 Drinking Water Survey | 2020 | Nutrition | 158 with assumptions | Altman, Emily | Schools (all levels) | 10 | 6 |
| Food Service Manager Questionnaire | 2020 | Nutrition | 30 | Au, Lauren | ES and MS | 12 | 2 |
| School Policies and Practices for Nutrition and Physical Activity | 2020 | Nutrition and Physical activity | 44 | Au, Lauren | ES and MS | 12 | 2 |
| CTPR School Tobacco Policy Index | 2013 | Tobacco/Drug | 40 | Barbero, Colleen | School Districts | 14 | 4 |
| Bassi School Policy Assessment | 2019 | Wellness Policy | 98 | Bassi, Shalini | Schools (all levels) | 10 | -1 |
| Bassi School Environment Assessment Observational Checklist | 2019 | Wellness Policy | 146 | Bassi, Shalini | Schools (all levels) | 10 | 0 |
| School Environment and Policy Survey | 2009 | Nutrition | 54 | Belansky, Elaine S | ES | 10 | 0 |
| School Environment and Policy Survey | 2009 | Physical Activity | 32 | Belansky, Elaine S | ES | 11 | 0 |
| School Environment and Policy Survey | 2009 | Wellness Policy | 90 | Belansky, Elaine S | ES | 11 | 0 |
| School Wellness Policy Implmentation Questionnaire | 2012 | Wellness Policy | 29 | Budd, Elizabeth | HS | 11 | 0 |
| Gaines Policy Grade Checklist | 2011 | Wellness Policy | 12 | Gaines, Alisha | School Districts | 13 | 0 |
| Gaines School Wellness Survey | 2011 | Wellness Policy | 60 | Gaines, Alisha | School Districts | 10 | 0 |
| Galan school board questionnaire | 2012 | Tobacco/Drug | 14 | Galan, Iñaki | MS and HS | 12 | 0 |
| Gillies Parent Survey | 2020 | Nutrition | 20 | Gillies, Christina | Schools (all levels) | 12 | 0 |
| 2014SHProfile-HETeacherSurvey | 2019 | Health Education | 214 | Guerra, Laura | MS and HS | 13 | 4 |
| 2014SHProfile-Principal Survey | 2019 | Wellness Policy | 179 | Guerra, Laura | MS and HS | 11 | 4 |
| Harvey SWP survey | 2018 | Wellness Policy | 32 | Harvey, Susan | School Districts | 12 | 0 |
| Heinze smoke-free schools survey | 2019 | Tobacco/Drug | NR | Heinze, Clara | HS | 10 | 6 |
| School Health Policies & Practices Questionnaire | 2013 | Wellness Policy | 288 | Hood, Nancy | MS and HS | 7 | 4 |
| Global Youth Tobacco Survey | 2013 | Tobacco/Drug | 54 | Huang, Song-Lih | MS | 13 | 5 |
| 2008 School Health Profile - School Principal Questionnaire | 2015 | Wellness Policy | 156 | Kehm, Rebecca | MS and HS | 9 | 4 |
| Monitoring the Future Principal Tobacco Survey | 2005 | Tobacco/Drug | 154 | Kumar, Revathy | HS | 9 | 6 |
| Observational School Environment Checklist | 2018 | Wellness Policy | 44 | Lane, Hannah | ES and MS | 13 | 0 |
| Perceptions of Environment at School | 2018 | Wellness Policy | 40 | Lane, Hannah | ES and MS | 12 | 0 |
| Teacher Awareness and Implementation of Food and Physical Activity Policies Survey | 2012 | nutrition and physical activity | 27 | Lanier, William | ES | 12 | 0 |
| Policy Implementation Questionnaire | 2020 | Wellness Policy | 37 | Levay, Adrienne | Schools (all levels) | 11 | 0 |
| Wellness policy implementation | 2009 | Wellness Policy | 77 | Longley | School Districts | 10 | 4 |
| Wellness Policy Evaluation Tool | 2015 | Wellness policy | 96 | Lucarelli, Jennifer F. | MS | 11 | 1 |
| Healthy, Hunger-Free Kids Act Survey | 2018 | Nutrition | 33 | Mansfield, Jennifer | School Districts | 12 | 1 |
| McDonnell Wellness Policy | 2008 | Wellness Policy | 45 | McDonnell | Schools (all levels) | 12 | 0 |
| McIsaac School Food Environment survey | 2019 | Nutrition | 144 | McIsaac, Jessie-Lee | Schools (all levels) | 10 | 0 |
| Local School Wellness Policy Principal Survey | 2011 | Wellness Policy | 155 | Molaison, Elaine | Schools (all levels) | 10 | 0 |
| Molaison Attitudes of School Professionals and Parents in Elementary Schools | 2008 | Wellness Policy | NR | Molaison | Schools (all levels) | 9 | 0 |
| Octaria school nutrition policy survey | 2020 | Nutrition | 25 | Octaria, Yessi | MS and HS | 13 | 0 |
| Orava Food Environment Scan | 2017 | Nutrition | 66 | Orava, Taryn | Schools (all levels) | 10 | 0 |
| Healthy School Planner Eating Detailed Questionnaire | 2017 | Nutrition | NR | Orava, Taryn | Schools (all levels) | 13 | 0 |
| Principles of Effectiveness | 2002 | Tobacco/Drug | 17 | Pankratz, M | School Districts | 11 | 3 |
| Adoption of Principles of Effectivess | 2002 | Tobacco/Drug | 8 | Pankratz, M | School Districts | 11 | 0 |
| Patel Observation Tool Drinking Water Access | 2012 | Nutrition | 91 | Patel, Anisha | Schools (all levels) | 9 | 0 |
| Patel 2012 Drinking Water Policy Survey | 2012 | Nutrition | 28 | Patel, Anisha | Schools (all levels) | 11 | 0 |
| Pettigrew_Health Food and Drink Policy Survey | 2018 | Nutrition | 119 | Pettigrew, Simone | Schools (all levels) | 9 | 0 |
| Phillips_Adolescent | 2013 | Wellness Policy | 77 | Phillips, Martha | MS and HS | 10 | 0 |
| Probart_School Wellness | 2010 | Wellness Policy | 62 | Probart | Schools (all levels) | 10 | 1 |
| Reilly canteen manager survey | 2019a | Nutrition | 46 | Reilly, Kathryn | MS and HS | 11 | 0 |
| Quick Menu Audit | 2019b | Nutrition | 84 | Reilly, Kathryn | MS and HS | 13 | 0 |
| Reynolds Sun Safe Schools Teacher Survey | 2020 | Sun Safety | 72 | Reynolds, Kim | ES | 11 | 0 |
| Reynolds Sun Safe Schools Principal Survey | 2020 | Sun Safety | 64 | Reynolds, Kim | ES | 11 | 0 |
| Reynold Sun Safe Schools Policy Coding | 2020 | Sun Safety | 10 | Reynolds, Kim | ES | 13 | 0 |
| Rozema_Outdoor Smoking Ban | 2017 | Tobacco/Drug | 14 | Rozema, A. D. | MS and HS | 12 | 3 |
| Schreuders smoke free school policy survey 2019 | 2019 | Tobacco/Drug | 62 | Schreuders, Miachael | HS | 10 | 0 |
| School Nutrition and Physical Activity Practices Survey | 2012 | nutrition and physical activity | 40 | Schwartz, Marlene | Schools (all levels) | 11 | 0 |
| School Health Profiles Principal Survey | 2017 | Wellness Policy | 178 | Snelling, Anastasia | ES and MS | 11 | 2 |
| School Health Profiles Teacher Survey | 2017 | Wellness Policy | 170 | Snelling, Anastasia | ES and MS | 12 | 2 |
| SNAP Policy Checklist | 2011 | Wellness Policy | 79 | Taylor, Jennifer | ES | 10 | 0 |
| FC Questionnaire | 2018 | Nutrition | 35 | Theodore | ES | 17 | 0 |
| Vecchiarelli Soda & Junk Food Ban Survey | 2006 | Nutrition | 45 | Vecchiarelli, Stephanie | HS | 12 | 0 |
| Team Nutrition Questionnaire (modified from SHI) | 2012 | Wellness Policy | 70 | Wall, Rachel | School Districts | 15 | 1 |
| Watts School Policy Physical Activity and Nutrition Survey | 2014 | nutrition and physical activity | 248 | Watts, Allison | Schools (all levels) | 11 | 3 |
| Webster PA Classroom Survey | 2013 | Physical Activity | 39 | Webster, Collin | ES | 10 | 2 |
| Healthy Canteen Policy Survey | 2017 | Nutrition | 9 | Wolfenden, Luke | ES | 11 | 1 |

Note: PAPERS = Psychometric and Pragmatic Evidence Rating Scale

1. Henrikson NB, Blasi PR, Dorsey CN, Mettert KD, Nguyen MB, Walsh-Bailey C, et al. Psychometric and Pragmatic Properties of Social Risk Screening Tools: A Systematic Review. Am J Prev Med. 2019;57(6S1):S13-S24.

2. Lewis CC, Mettert KD, Dorsey CN, Martinez RG, Weiner BJ, Nolen E, et al. An updated protocol for a systematic review of implementation-related measures. Syst Rev. 2018;7(1):66.

3. Powell BJ, Stanick CF, Halko HM, Dorsey CN, Weiner BJ, Barwick MA, et al. Toward criteria for pragmatic measurement in implementation research and practice: a stakeholder-driven approach using concept mapping. Implement Sci. 2017;12(1):118.

4. Stanick CF, Halko HM, Nolen EA, Powell BJ, Dorsey CN, Mettert KD, et al. Pragmatic measures for implementation research: development of the Psychometric and Pragmatic Evidence Rating Scale (PAPERS). Translational Behavioral Medicine. 2019.

5. Glasgow RE. What does it mean to be pragmatic? Pragmatic methods, measures, and models to facilitate research translation. Health Educ Behav. 2013;40(3):257-65.
